# Causes of Colectomy in Patients with Ulcerative Colitis: Findings from an Iranian National Registry

**DOI:** 10.34172/aim.28887

**Published:** 2024-07-01

**Authors:** Zahra Momayez Sanat, Homayoon Vahedi, Reza Malekzadeh, Amir Kasaeian, Negar Mohammadi Ganjaroudi, Alireza Sima, Fariborz Mansour Ghanaei, Mohammadreza Ghadir, Hafez Tirgar Fakheri, Siavosh Nasseri Moghaddam, Sudabeh Alatab, Anahita Sadeghi, Amir Anushiravani, Iradj Maleki, Abbas Yazdanbod, Hassan Vossoughinia, Mohammadreza Seyyedmajidi, Sayed Jalaleddin Naghshbandi, Nadieh Baniasadi, Baran Parhizkar, Saied Matinkhah, Shahsanam Gheibi, Roya-sadat Hosseini Hemmat Abadi, Seyedmohamad Valizadeh Toosi

**Affiliations:** ^1^Digestive Disease Research Center, Digestive Disease Research Institute, Tehran University of Medical Sciences, Tehran, Iran; ^2^Digestive Oncology Research Center, Digestive Diseases Research Institute, Shariati Hospital, Tehran University of Medical Sciences, Tehran, Iran; ^3^Research Center for Chronic Inflammatory Diseases, Shariati Hospital, Tehran University of Medical Sciences, Tehran, Iran; ^4^Clinical Research Development Unit, Shariati Hospital, Tehran University of Medical Sciences, Tehran, Iran; ^5^School of Medicine, Shahid Beheshti University of Medical Sciences, Tehran, Iran; ^6^Sasan Alborz Biomedical Research Center, Masoud Gastroenterology and Hepatology Center, Tehran, Iran; ^7^Gastrointestinal and Liver Diseases Research Center, Guilan University of Medical Sciences, Guilan, Iran; ^8^Gastroenterology and Hepatology Diseases Research Center, Qom University of Medical Science, Qom, Iran; ^9^Gut and Liver Research Center, Non-communicable Diseases Institute, Mazandaran University of Medical Sciences, Sari, Iran; ^10^Digestive Diseases Research Center, Ardabil University of Medical Sciences, Ardabil, Iran; ^11^Department of Gastroenterology and Hepatology, Mashhad University of Medical Sciences, Mashhad, Iran; ^12^Golestan Research Center of Gastroenterology, Golestan University of Medical Science, Gorgan, Iran; ^13^Liver and Digestive Research Center, Research Institute for Health Development, Kurdistan University of Medical Sciences, Sanandaj, Iran; ^14^Noncommunicable Diseases Research Center, Bam University of Medical Sciences, Bam, Iran; ^15^Isfahan University of Medical Sciences, Isfahan, Iran; ^16^Maternal and Childhood Obesity Research center, Urmia University of Medical Sciences, Urmia, Iran; ^17^Shahid Sadoughi University, Yazd, Iran

**Keywords:** Colectomy, Dysplasia, Ulcerative colitis

## Abstract

**Background:**

Ulcerative colitis (UC) is a form of inflammatory bowel disease (IBD) marked by rectal and colon inflammation, leading to relapsing symptoms. Its prevalence is increasing, particularly in developed nations, impacting patients’ health. While its exact cause remains unclear, genetic and environmental factors are implicated, elevating the risk of colorectal cancer (CRC). Colectomy, though declining, is still performed in select UC cases, necessitating further study.

**Methods:**

We analyzed data from the Iranian Registry of Crohn’s and Colitis (IRCC) to examine UC patients undergoing colectomy. We collected demographic and clinical data from 91 patients, focusing on dysplasia. Statistical analyses assessed dysplasia risk factors.

**Results:**

Patients with dysplasia were older at diagnosis and surgery compared to those without dysplasia. Age emerged as a significant risk factor for dysplasia in UC patients undergoing colectomy. No significant associations were found between dysplasia and other factors.

**Conclusion:**

Age plays a crucial role in dysplasia risk among UC patients undergoing colectomy. Older age at diagnosis and surgery may indicate a higher risk of dysplasia and CRC. Clinicians should consider age when managing UC patients and implementing screening protocols. Further research with larger samples is needed to confirm these findings.

## Introduction

 Ulcerative colitis (UC) stands as a distinctive subtype within the spectrum of inflammatory bowel diseases (IBD), characterized by initial involvement of the rectum, often progressing contiguously to affect the colon.^1^ Lesions characteristic of UC manifest superficially, with inflammation predominantly confined to the mucosal and submucosal layers.^2^ UC occurs in a relapsing and remitting form.^3^ Patients with UC can present with intestinal manifestations such as bloody diarrhea, abdominal pain, tenesmus, and fecal urgency, or extra-intestinal symptoms such as musculoskeletal, cutaneous, and hepatobiliary manifestations.^4,5^

 Over recent years, the prevalence of UC has witnessed a surge, with North America and Europe registering the highest prevalence rate.^6-8^ Moreover, increased prevalence of UC has been reported in industrialized countries.^9,10^ Previous studies have reported UC prevalence and incidence ranging from 7.6 to 245 and 1.2 to 20.3 cases per 100 000 individuals per year, respectively.^11-14^ The exact etiologies of UC are unknown, but it is believed that genetics play an essential role in the development of the disease.^15^ Reports indicate a four-fold higher risk of UC among first-degree relatives of affected individuals.^14^ Additionally, environmental factors exert significant influence on UC progression, with diet, certain medication use, vitamin D levels, stress, and smoking history implicated as potential risk factors.^16^ UC is associated with an increased risk of malignancies.^17^ Estimates suggest a progressive increase in colorectal cancer (CRC) risk with disease duration, reaching 2%, 8%, and 18% at 10, 20, and 30 years, respectively.^18^ Notably, CRC accounts for a significant proportion (10‒15%) of mortality in IBD cases.^19^ Risk factors for CRC in UC patients include disease extent, duration, concurrent primary sclerosing cholangitis, and family history of CRC.^18,20-22^

 Although the development of new biologic therapies has reduced the need for surgical procedures, a significant number of patients still undergo operations such as colectomy.^23-28^ Indications for colectomy include failure of medical therapy, increased risk of cancer, fulminant colitis, toxic megacolon, and intensive extra-intestinal manifestations.^29^

 The Iranian Registry of Crohn’s and Colitis (IRCC) is a multicenter prospective registry that aims to provide a better understanding of the natural history, phenotype, treatment response, complications, and survival of IBD patients in Iran.^30^ The colectomy data from this registry have not been reported previously. In this regard, in the current study, we retrospectively examined the baseline characteristics and the presence of identified risk factors for colectomy among all UC patients in the IRCC database.

## Materials and Methods

###  Study Design 

**Figure 1:**
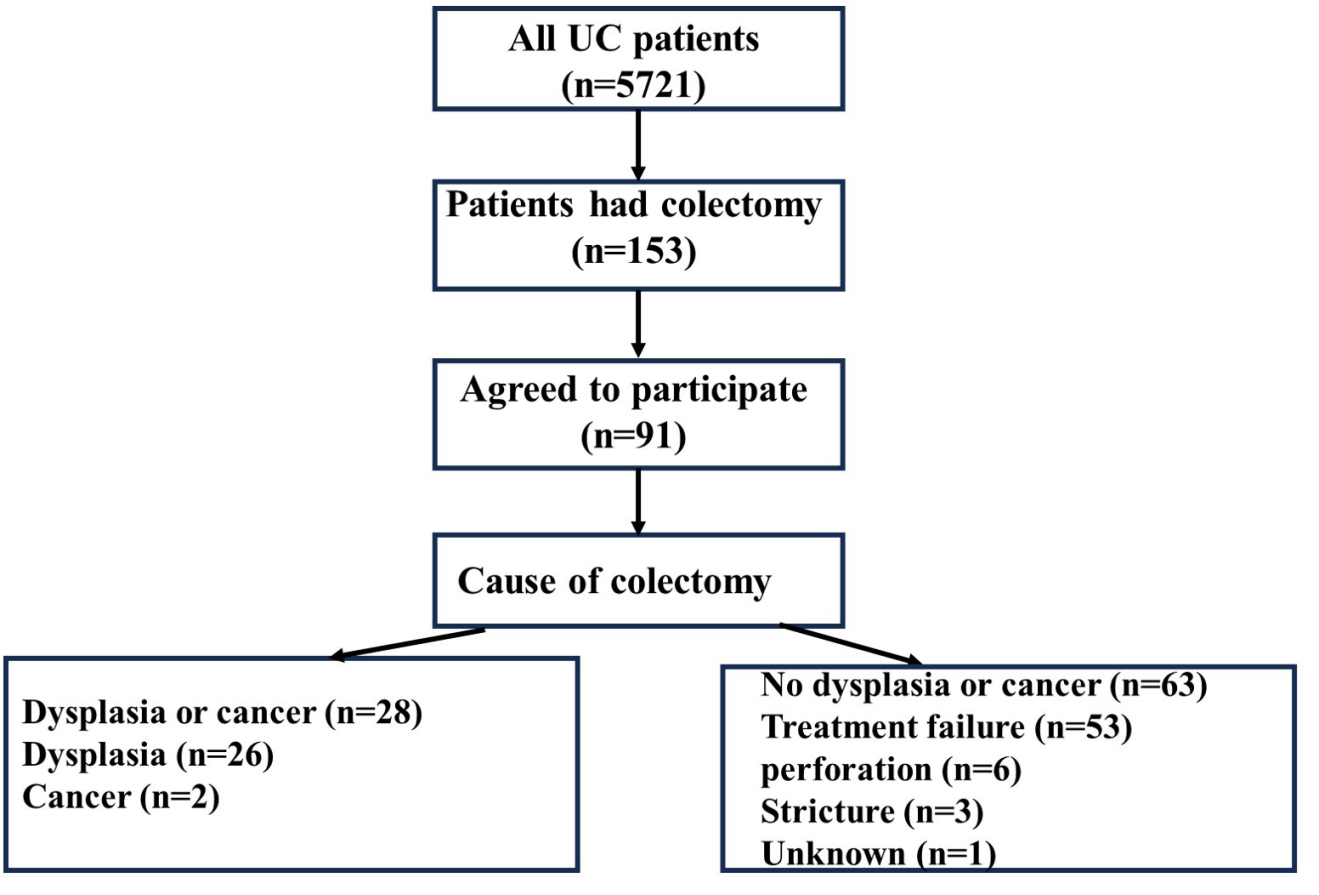
Flowchart of the included patients

 The present study is a cross-sectional, national registry-based study that includes all patients with an established UC diagnosis who were registered in IRCC. UC diagnosis was based clinical, radiological, colonoscopic, and pathologic findings according to international guidelines for IBD diagnosis. The detail of IRCC has been published elsewhere.^30^ In this registry, a total of 5721 UC patients were enrolled between 2017 and 2022. Among them, 153 patients underwent colectomy, with 91 of these individuals consenting to participate in the present study (Figure 1).

###  Study Participation and Data Collection

 This study targeted UC patients aged 18 or higher who had undergone colectomy regardless of reason. After obtaining informed consent from the patients, demographic and clinical characteristics were collected from medical records. The collected information included demographic characteristics, disease extent, extra-intestinal manifestations, IBD medication history, cause of colectomy, and type of colectomy (elective vs emergency).

###  Statistical Analyses

 Statistical analysis was performed using the Statistical Package for the Social Sciences (SPSS) version 16. Descriptive statistics, including mean and standard deviation, were utilized for reporting quantitative variables. The chi-square test was employed for categorical variables, while the independent *t *test was used for continuous variables. Univariate logistic regression was conducted to assess differences between the two groups. Statistical significance was set at *P* < 0.05.

## Results

**Figure 2:**
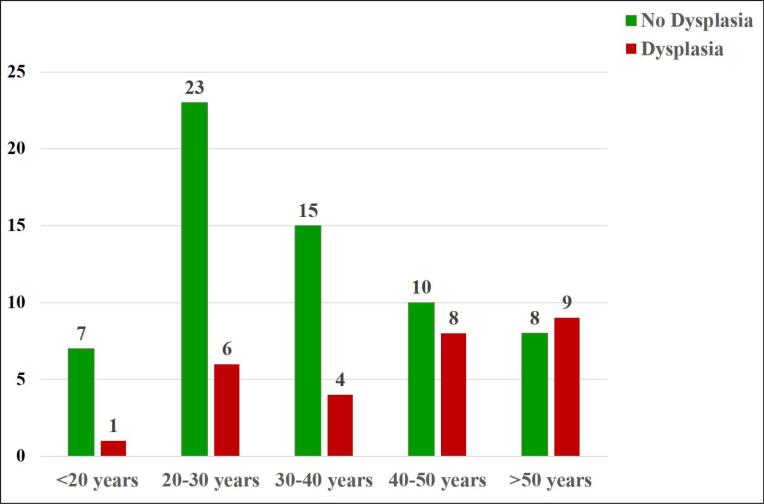
Age at diagnosis

 The patients’ flowchart, illustrated in Figure 1, outlines the enrollment process, culminating in the inclusion of 91 UC patients in our study. Among these participants, 49 (55.6%) were male. Of the 91 patients admitted, 28 were diagnosed with cancer or dysplasia, while 63 were not. Among the 63 patients whose colectomy was not attributed to dysplasia, 53 (83.9%) experienced treatment failure, 6 (9.4%) encountered perforation, 3 (4.6%) developed strictures, and 1 (1.2%) had an indeterminate cause. The mean age of participants was 54.3 ± 13.6 years in the dysplasia group and 43.6 ± 12.1 years in the non-dysplasia group. Table 1 presents the demographic characteristics of the two study groups based on cause of colectomy (dysplasia vs non-dysplasia), while Figure 2 delineates the age at diagnosis distribution of the two study groups.

**Table 1:** Baseline Characteristics of the Study Population

**Variable**		**All Cases with Colectomy (n=91)**		***P *** **Value**
		**No Dysplasia (n=63)**	**Dysplasia (n=28)**	
Age	< 55 years	51	15	< 0.001
	> 55 years	12	13	
BMI	Mean (SD)	24.5 (3.4)	25.7 (3.1)	0.35
Age at diagnosis	Mean (SD)	29.2 (12.1)	36.2 (14.1)	< 0.001
Duration of disease	Mean (SD)	14.5 (8.6)	18.1 (8.7)	0.077
Gender	Male	33	17	0.46
	Female	30	11	
Marital status	Single	10	3	0.74
	Married	52	25	
Education level	Less than 12 years	8	9	0.09
	12 years	32	11	
	More than 12 years	23	8	
Ethnicity	Persian	44	24	0.43
	Kurd	1	1	
	Lor	6	1	
	Turk	11	2	
	Baluch	1	0	
Blood group	A	19	7	0.66
	AB	3	1	
	B	14	6	
	O	20	9	
	Unknown	6	4	
Extension of disease	Left colitis	4	2	0.96
	Pancolitis	56	23	
	Unknown	2	1	
UC diagnosis	Inpatient	8	4	0.83
	Outpatient	55	24	
Type of colectomy	Elective	57	26	0.99
	Emergent	6	2	
Smoking	Yes	9	7	0.36
	No	54	21	
Opium use	Yes	1	1	0.55
	No	62	27	
Alcohol use	Yes	3	1	0.81
	No	60	27	
History of blood transfusion	Yes	45	12	0.12
	No	18	16	
History of CMV colitis	Yes	2	0	0.34
	No	61	28	
Family history of UC	Yes	21	7	0.42
	No	42	21	

Table 2 delineates the pharmacological regimens administered to the patients. Interestingly, no significant difference was observed between patients whose initial treatment involved biological agents and those who received other medications. Furthermore, no statistically significant association was found between the duration of medication use and the occurrence of dysplasia. Mesalazine emerged as the most commonly prescribed initial drug, with a notable proportion of patients with dysplasia having received this medication. Among those who progressed to the second and third stages of drug therapy, none of the six individuals treated with Infliximab were diagnosed with dysplasia. Conversely, only one out of fifteen patients who received Azathioprine exhibited dysplasia. The primary reason for transitioning from the initial drug regimen was treatment failure in 44 patients, while adverse drug reactions prompted a change in medication for 11 individuals, and non-compliance was cited as the reason in 2 cases.

**Table 2:** Treatment Steps of the Study Population

**Steps of Treatment**	**Dysplasia**	**No Dysplasia or Cancer**
First		
Azathioprine	2	0
Mesalazine	52	20
Sulphasalazine	8	5
Second		
Azathioprine	10	1
CinnoRA®	4	4
Infliximab	5	0
Mesalazine	5	3
Sulphasalazine	8	1
Third		
Azathioprine	2	0
CinnoRA®	5	1
Infliximab	1	0

Table 3 show the extra-intestinal manifestations in the two groups. Statistical analysis showed no statistically significant difference between the two groups in any of the extra-intestinal manifestations.

**Table 3:** Comparison of Extra-intestinal Manifestations in the Two Groups

**Extra-Intestinal Manifestation**	**Dysplasia (n=28)**	**No Dysplasia (n=63)**	***P *** **Value**
Articular disease	1 (3.8)	2 (7.6)	0.941
Autoimmune hepatitis	1 (3.8)	0 (0.0)	0.248
Ophthalmologic disease	1 (3.8)	3 (4.8)	0.857
Primary sclerosing cholangitis	2 (7.6)	2 (3.2)	0.578
Perianal abscess	0 (0.0)	1 (1.6)	0.928

 According to the findings derived from the univariate analysis, as depicted in Table 4, several factors were found to be significantly correlated with an increased risk of dysplasia or cancer in UC patients undergoing colectomy. Notably, age (OR = 1.06), age at the time of diagnosis of the disease (OR = 1.04), and age at the time of surgery (OR = 1.06) demonstrated statistically significant associations with the heightened risk of dysplasia or cancer.

**Table 4:** Results of Univariate Analysis

**Factor**	**Odds Ratio**	**95 % CI**	***P *** **Value**
Age	1.06	1.02‒1.11	0.001
Age at diagnosis	1.04	1.00‒1.08	0.019
Age at surgery	1.06	1.02‒1.11	0.002
Gender (Male/Female)	1.40	0.55‒3.57	0.475
Duration of disease	2.61	0.98‒6.78	0.07
Marital status (Married/Single)	1.47	0.37‒5.86	0.581
Education Level	0.87	0.73‒1.03	0.123
Body mass index	1.11	0.96‒1.28	0.135
Smoking (Yes/No)	1.76	0.55‒5.60	0.361
Non steroid anti- inflammatory drugs (Yes/No)	1.25	0.41‒3.78	0.774

 However, other demographic and clinical variables, including gender (OR = 1.40), marital status (OR = 1.47), duration of disease (OR = 2.61), education level (OR = 0.87), BMI (OR = 1.11), smoking (OR = 1.76), and non-steroidal anti-inflammatory drugs, did not exhibit a significant association with dysplasia in UC patients undergoing colectomy.

## Discussion

 UC is a subgroup of IBD, which causes inflammation, production of reactive oxygen species (ROS), and increased epithelial cell turnover, resulting in low-grade dysplasia, high-grade dysplasia, and consequently CRC.^31-33^ The relationship between UC and CRC has been established in previous studies.^18,34,35^ The prevalence of CRC in UC patients was reported at 3.7%^36^; however, in our study, the prevalence of colorectal dysplasia and cancer was 31.1%. In one meta-analysis, the incidence of CRC in UC patients was reported at 1.58 per 1000 patients per year.^37^ In a meta-analysis by Eaden et al, the incidence of CRC in UC patients was reported at 2%, 8% and 18% in 10 years, 20 years and 30 years, respectively.^38^ As a result of the occurrence of CRC, UC patients should be followed up based on surveillance protocols, although with the advent of new therapies including biologic agents, the risk of CRC in UC patients has declined.^39-41^ If malignant lesions are found in the follow-up tests, surgery is considered an option.^28,29^ Another indication for surgery in UC patients is when patients are not responding to pharmacological treatment.^29,42^ Although with the advent of new therapies and treatment protocols, the need for surgery has declined, a significant number of patients undergo surgery.^42-44^

 In this study, our objective was to assess the risk factors of dysplasia in UC patients who underwent colectomy. We evaluated 12 factors, among which three factors showed significant associations with an elevated risk of neoplasia in UC patients: patient’s age, age at the time of diagnosis, and age at the time of surgery.

 Based on the results of this study, UC patients who underwent colectomy due to dysplasia were significantly older than those who underwent colectomy due to other reasons. Moreover, they were significantly older at the time of diagnosis and surgery. This finding implies a higher chance of CRC in older UC patients than young ones. The results of other studies are conflicting. In a cohort study on UC patients, Gyde et al reported that the highest chance of cancer in a UC patient is expected to occur at around 50.^45^ In another retrospective study by Karvellas et al, UC patients diagnosed after the age of 40 were at higher risk of CRC than UC patients diagnosed before the age of 40.^34^ Bamba and Nishiyama showed the higher risk of CRC in elderly UC patients.^46^ The increased cancer risk in older patients can be attributed to lead-time bias and pathological processes. The chance of undiagnosed colitis for a longer duration is higher in older UC patients.^34^ Moreover, carcinogenesis processes develop at advanced ages, like DNA hypermethylation which has a potential role in colonic neoplasia.^47^

 Our study revealed no significant association between blood group and the risk of dysplasia in UC patients. This finding aligns with research conducted by Al-Sawat, which found no statistical difference in the relationship between blood group types and CRC risk.^48^ Similarly, Khalil et al observed no significant difference between blood group types and the risk of CRC in their study.^49^

 In our study, the ethnicity of the patients was not a risk factor for occurrence of dysplasia in UC. Ethnicity can play an important role in the development of UC.^12,50-52^ Studies showed that ethnicity can also affect the incidence and prevalence of CRC.^53,54^ In a study by Damas et al, the prevalence of IBD-related dysplasia was significantly affected by ethnicity.^55^

 In our study, we did not find smoking to be a significant risk factor for dysplasia in UC patients. However, our sample size was small, so caution is advised in interpreting these results. The impact of smoking on UC remains controversial, with current smoking being potentially protective while former smoking may pose a risk.^56^ Smoking is thought to be a risk factor for CRC. A recent meta-analysis showed former smokers, and current smokers to be at higher risk of CRC.^57^ In a recent retrospective cohort study, former smokers were at increased risk of colorectal neoplasia in the UC population, while passive smoking had no significant effect.^58^ Due to the divergent impact of smoking on the UC severity and risk of CRC, we suggest further studies to assess the pooled results of cigarette smoking on CRC among UC patients.

 In this study, positive family history of UC was not significantly associated with increased risk of CRC in UC patients. This finding is consistent with the result of a cohort study by Askling et al in which although a positive family history of CRC was associated with increased risk of CRC in the IBD population, no significant relation was found for family history of IBD.^22^

 Our study did not find a significant difference in the prevalence of extra-intestinal manifestations of UC between patients with cancer and those without, contrary to previous findings indicating an increased cancer risk in IBD patients with extra-intestinal manifestations.^59^ We believe the small sample size of our study is one of the reasons which necessitate the result to be considered with caution. In addition, data of some patients were not available. We recommend more high-quality, studies with larger sample size and in multicenter settings to assess the relationship between the extra-intestinal manifestations of IBD and CRC.

## Conclusion

 In summary, we discovered a correlation between dysplasia and advanced age at diagnosis and operation. According to these results, older UC patients may be at increased risk of CRC. It is recommended that clinicians treat certain patient groups as high-risk individuals and that specific screening and surveillance strategies are implemented for them.
